# Mental disorders are not brain disorders

**DOI:** 10.1111/jep.12048

**Published:** 2013-05-21

**Authors:** Natalie F Banner

**Affiliations:** Centre for the Humanities and Health, King's College LondonLondon, UK

**Keywords:** Jaspers, mental disorder, psychiatric classification, psychiatry, psychological realism, stigma

## Abstract

As advances in neuroscience and genetics reveal complex associations between brain structures, functions and symptoms of mental disorders, there have been calls for psychiatric classifications to be reconfigured, to conceptualize mental disorders as disorders of the brain. In this paper, I argue that this view is mistaken, and that the level at which we identify mental disorders is, and should be, the *person*, not the brain. This is not to deny physicalism or argue that the mental realm is somehow distinct from the physical, but rather to suggest the things that are going ‘wrong’ in mental disorder are picked out at the person-level: they are characterized by breaches in epistemic, rational, evaluative, emotional, social and moral norms. However, as our scientific understanding of the brain becomes advanced, what makes an identified neurobiological difference in brain structure or functioning indicative of pathology is its association with these behaviours at the person-level. Instead of collapsing psychiatry into biomedicine, biomedicine may benefit from drawing closer to the expertise of psychiatry, as it is able to accommodate social, psychological and biological explanations while focusing on the person, within their environment.

## Introduction

The relation between the mental and the physical, and between mental and physical disorders, has a long, complex and contentious history, both in the philosophy of mind and science and in conceptualizing the nature and role of medicine. In this paper, I want to skip over some of the more profound philosophical questions about the nefariously tricky relation between mind and body, mental causation and the nature of mental content.^1^

Instead, I focus on a conceptual question with clear practical consequences: are mental disorders, disorders of the brain? To do this, I first consider why this distinction matters, and highlight some areas of difference between mental and physical disorders. I will put forward an argument that mental disorders ought not to be thought of as disorders of the brain, and consider some implications that follow if they are conceptualized as brain disorders. I am largely in agreement with White's diagnosis of the problem 1, which is, in brief, that psychiatric classifications reflect an anachronistic division between mind and brain fail to keep up with current scientific understanding of mental disorders and are highly stigmatizing. However, I disagree that the prescription for the cure is to conceptualize mental disorders as disorders of the brain.

## Mind or brain: why does the distinction matter?

The classification of mental disorders is a contentious area, and it is important to understand that debates over the validity, vocabulary and conceptualizations of mental disorder are not merely the academic and theoretical concerns of philosophers and nosologists. Although primarily an epidemiological and research tool, coding in the major systems of classification determines how a psychiatric problem is clinically approached, the treatments available to patients and, in insurance-based health care systems such as that in the USA, determine what treatments can be paid for. Classifications are intended to divide up the landscape of mental disorders in empirically valid and clinically reliable way. Perhaps more so than with any other type of illness, how their condition is defined can be an extremely important issue for patients with a mental disorder diagnosis: in no other domain of medicine is there a vociferous and substantial survivor and service-user movement critical of the way their disorders are classified, and patients treated. One of the most prominent issues of recent times is the extent to which mental disorders can and ought to be classified as medical conditions, as opposed to, for example, problems in living 2. The question of whether mental disorders are disorders of the brain represents one aspect of this conceptual issue for classification, which has long, deep political and social implications.

Such questions in psychiatry are not new. The year 2013 is the centenary of the publication of Karl Jaspers' *General Psychopathology* [Bibr b3] and psychiatry is now finding itself a subject to similar debates and controversies as those which dogged the field 100 years ago 4. Jaspers wrote that text in response to what he considered to be an unhelpful biological reductionism that accompanied rapid advances in medical understanding of the brain in the late 19th and early 20th centuries. Around that time, the eminent neurologist and psychiatrist Wilhelm Griesinger had insisted that *‘all mental illnesses are cerebral illnesses’* 5 and this position has recently been gaining currency within the profession once more. However, Jaspers argued that *‘there has been no fulfilment of the hope that clinical observation of psychic phenomena, of the life-history and of the outcome might yield characteristic groupings which would subsequently be confirmed in the cerebral findings*’ ([Bibr b3], p.568). It is this theme I wish to pick up on in this paper; to consider the relation between disorders, we identify through their physical pathology, and those which, I will argue, we do not and indeed should not, whatever advances in neuroscience may reveal about the complex workings of the brain.

Psychiatry is fundamentally different from medical disciplines such as cardiology or oncology, and we can perfectly and consistently hold this view without falling prey to accusations of Cartesian dualism or claims that mental illness is ‘all in the mind’. One can legitimately hold both that ultimately, the mind is made up of brain stuff, and that mental disorders are not reducible to brain disorders. To understand this, let us consider how physical and mental disorders are identified.

## Physical and mental disorders

In the case of physical conditions, what picks out a condition as being a disorder is that there is presumed or found to be some dysfunction (howsoever construed, whether biostatistically (e.g. Boorse 6) or evolutionarily (e.g. Wakefield 7) as deviation from a norm of functioning in the body. More recent models have advanced an actuarial, risk-based model of disease 8 but on both kinds of account, the problem is thought to lie in the internal workings of the biological body.^2^ Across the straightforwardly physical realm, there are three main divisions in the classification of how this internal failing or dysfunction is characterized. Some disorders are grouped according to known aetiology, for example cancers or genetic conditions. Others are grouped pragmatically, by the medical specialization most appropriately suited to treating them. Conditions treated in obstetrics and geriatrics are two such examples. Thirdly, some disorders are classified by the organ system affected, for example respiratory disorders or those of the gastrointestinal tract. Neurology falls under this heading, treating disorders of the central nervous system.

By contrast, in the mental case, what is picked out as disordered or problematic is that there is a disturbance of some kind in the person's thoughts, feelings or behaviours. Classificatory systems such as the DSM-IV and ICD-10 identify mental disorders as patterns of behavioural and psychological states associated with distress, disability, risk of suffering or a significant loss of freedom (along with other criteria). The predominant neo-Kraeplinian classification systems categorize mental disorders as clusters of self-reported and behavioural signs and symptoms, with no implication in terms of underlying aetiology or physical process: this provides a basis from which to initiate subsequent physical investigation 10. Criteria for classification are thus observational and not themselves indices of biological or cognitive functioning. However, arguments, such as White *et al*. ‘s 1 have recently been put forward that our understanding of the scientific basis of mental disorders is advancing enough for classification to undergo a radical shift to categorize according to the dysfunction or deviation occurring in the known organ affected: the brain. I suggest that this is a mistake, for two reasons. Firstly, despite significant technological progress, it should not be assumed that specific brain pathologies or deviations in functioning will be found that inform our understanding and classification of mental disorders. Secondly, and more importantly, classifying according to signs and symptoms such as patterns of thought, expression and behaviour retains the use of person-level concepts and thus better reflects the nature of the disorders themselves. I will address these reasons in turn.

## Pathologies in the brain?

There have been many astonishing advances in our understanding of neuroscience, of genetics and epigenetics, and of neurodevelopment, but there is not a single identifiable biomarker for any mental disorder 11. Robust evidence suggests strong genetic components to many disorders that do not necessarily correspond with current classifications 12. Further developments in the science of the brain are highly likely to reveal heretofore unknown and surprising links between brain circuits, genetics, thought patterns and behaviour. A Nature editorial in 2010 13 deemed this current decade the decade for psychiatric disorders, and argued that our understanding of such disorders will be revolutionized by progress in genetics and neuroscience. For my present purposes, the interest in the increasing prevalence of natural science-based explanations is the implication that psychiatric disorders are identifiable as disorders of the brain, explained in terms of neurobiological *pathology*. The question I wish to press is this: should these technological and neuroscientific advances make a difference to how mental disorders are classified, identified, diagnosed and treated?

A prominent interpretation of the goals and methods of psychiatry suggests they should. The ‘strong’ interpretation of the medical model 14 leads either to *eliminitavism* about psychiatric categories (the belief that they will eventually be superseded by neurobiological categories) or *essentialism* (the view that psychiatry will and should be reduced to neurology through the matching of psychiatric categories to an underlying biological ‘essence’). On both of these views, disease and disorder should be understood in terms of morbid anatomy and physiology; eventually, it will be possible to generate causal hypotheses about psychiatric disorders in terms of neuropathology or dysregulation. Whether the key theoretical concepts come from cognitive neuroscience, molecular biology or some other basic brain science, the strong interpretation of the medical model assumes that explanations for mental disorders can be sought that cite pathogenic processes in brain systems, and that furthermore, future classifications should reflect this knowledge. Many different causal processes may operate, including social and psychological ones, but the strong interpretation relies on neurobiology and cognitive processes being fundamental, and for explanations of these varied causal processes to be reducible to the vocabulary of the brain sciences. If we are to be eliminativists or essentialists about mental disorder, this would have a significant and rapid impact on our classificatory systems: labels such as schizophrenia or bipolar disorder would be replaced, superseded perhaps, by classifications based on the presence or absence of a particular copy number variant, or a statistical variation in the structure or activity of a particular region of the brain.

It is worth taking a moment to consider whether this is a plausible view. Might there be a molecular or neurobiological reduction of the kind of symptoms and problems implicated in mental disorders? The notions of shame and guilt are central to depression 15; childhood abuse or neglect and socio-economic factors such as unemployment are powerful predictors of mental illness. Undoubtedly, these factors have effects in the brain, perhaps altering certain neural pathways from how they otherwise would have developed. However, this does not entail that there are identifiable *pathologies* of the brain associated with such complex sociocultural and psychological factors: ‘*They have brain effects, but the brain effects vary across classes of individuals in ways that depend on other environmental and genetic contexts’* 16. There is huge variation in the clinical presentations of every psychiatric disorder, and the ongoing challenge for psychiatry is to provide some method of grouping and classifying this diversity in a way that is useful both for diagnosis and treatment in the clinic, and for research. Even with a complete map of brain functioning and connectivity, there may be no underlying unified account of what's ‘wrong’ across different brains of people exhibiting similar symptoms, nor whether particular treatments are likely to affect brains (and people: an important distinction to acknowledge) in similar ways.

## Psychological realism

This brings us to the second reason for rejecting the conceptualization of mental disorders as brain disorders: what it is that identifies a person as suffering from a mental disorder in the first place? In some loose sense, in psychiatric disorders the person's *self* is affected, and the functional norms that he or she breaches are primarily epistemic (believing strange things), rational (thinking irrational thoughts, making incoherent connections), emotional (experiencing extremes of emotion that seems inappropriate or disproportionate to the situation), moral (doing or wanting things that contravene a societal moral code) or social (identified by others as being bizarre or objectionable) and those of self-knowledge (for example, experiencing problems with memory and intention). Thus the primary locus of disorder is among mental relationships, within a person's own psychological life, and in interpersonal relationships 17. In other words, the correct level of analysis for picking out that something is wrong, is the person, the self. The manifestation of the illness occurs at the level of observable behaviour and felt experience. Indeed, in no other medical discipline is the subjective experience of the patient quite so crucial to diagnosis and the aims of treatment.

*Whatever* the aetiology of the condition or its causal pathways in the brain – whether it is genetic, the product of gene-environment interactions, psychological, social or spiritual causes – the attribution of ‘disorder’ applies to the thoughts, feelings and behaviours of the person. Mental disorders are not mental disorders because of an assumed mental cause, but rather because they identify problems in the *person's* mental life and relationships. In this regard, I suggest that if we want to align psychiatry with one of the three main forms of categorization for physical disorders outlined above, we can think of current psychiatric nosology as based not on aetiology, nor on clinical pragmatism, but on the idea that the ‘organ’ affected is the *person,* within their environment. It would be a fallacy to suggest that the brain is the locus of disorder.

Many of the key features of psychiatric disorders can only be characterized and understood using the language of the mental realm. Establishing whether a given behaviour, thought pattern or emotional response is indicative of a disorder invokes precisely those concepts that reductionist accounts of psychiatry seek to eliminate or reinterpret in neurobiological terms: the psychological. This is not to deny that there is very likely to be a neurobiological basis to the behaviour, thought pattern or emotional response, and that the cause of these manifestations of pathology is biological. It is, rather, to argue that the level at which disorder can be identified is that of the *person*, who has both a brain, and a relevant social and environmental context. This view is called psychological realism, and it insists that whatever the technological and scientific advances we make in understanding the brain, its development and degeneration, we require psychological predicates in order to pick out instances of disorder 17.

It is of course true that many psychiatric disorders have clear physical symptoms: tiredness, pain, insomnia, changes in appetite. At the same time, physical disorders often have a strong psychological or emotional components or consequences: depression is commonly associated with Parkinson's; stress is strongly implicated in hypertension. None of this should give us undue cause for concern regarding our ontological commitments, or require us to defend a philosophically robust account of mental causation. At the level of ontology, of what exists in the world, one can be a physicalist but also hold that the ‘person’ level of analysis is conceptually irreducible to the stuff of the brain. It is important to remember that we are talking here about identifying the level at which disorder or dysfunction can be identified.

## The problems of locating mental disorders in the brain

Much research on the social determinants of health is revealing that environment has a causal impact on the functioning of the brain and body, but it also leads us to question where we should attempt to locate disorders themselves. Take, for example, a person who is a single parent on a run-down council estate, with a low-paid service job that provides little opportunity for meaningful interaction, control or fulfilment. He goes to his general practitioner (GP) complaining of fatigue, a sense of helplessness and hopelessness, loss of appetite and losing the enjoyment he used to feel when playing with his children. A diagnosis of depression would not be surprising under these circumstances, and a likely outcome from the GP appointment is a prescription for antidepressants and possibly an offer of a course of psychological therapy. Here, we *can* argue that the patient has a depleted level of serotonin in his brain, and a course of selective serotonin reuptake inhibitors may well help him to lift the black and bleak feelings that have descended upon him. But is this where we think the locus of disorder is? Is his *brain* malfunctioning in some respect?

I suggest that in focusing the attribution of disorder on the goings on in the brain, we are missing the essence of what is going ‘wrong’ for the individual attempting to get on with his or her life. This is not to say that biological explanations ignore or sideline social and psychological causes; quite the opposite, many attempts to develop biological explanations explicitly seek to account for the causative influences of social and psychological environment, through finding demonstrable impacts on brain function 1. Rather, it is to suggest that in championing the brain as the locus of disorder at the expense of the person-level, we are making a powerful, and in my view, mistaken, judgement about how the disorder is conceptualized and what kinds of approaches should be taken towards treatment. A recent controversial article in the New York Times sums up this problem well. Dr. Michael Anderson, a child psychiatrist in Georgia, frequently prescribes medication such as Ritalin and Adderall to children from poor socio-economic backgrounds who are struggling academically in inadequate, underfunded schools. He argues *‘we've decided as a society that it's too expensive to modify the kid's environment. So we have to modify the kid’* 18. His results in terms of improvements to academic grades are impressive and satisfactory for the parents of his patients, but there is an understandable feeling of unease associated with the idea that fixing the ‘problem’ via medication is a clinically or ethically justified solution.

Yet if mental disorders are conceptualized as brain disorders, with many and varied associated social and psychological causes, the conceptual primacy of the neurobiological would be deemed explanatorily satisfactory, and all causal pathways explained through their effect on the brain. The sheer complexity of the brain is one barrier to these kinds of explanations. But even if we did find strong evidence that there are specific, consistent, reliable neurobiological differences between those suffering from psychiatric disorders and those that do not, it is not clear that any such differences could be conceived as *pathological* without begging the question about what constitutes the disorder in the first place. What I mean by this is that neurobiological differences in themselves are not what ought to characterize disorder. There is much talk in the empirical literature about brain ‘abnormalities’, ‘deficits’ and ‘alterations’ associated with mental disorder symptoms. But conceptually, what marks these differences as indicative of disorder or dysfunction? It is the association they bear with deviations from epistemic, evaluative, emotional, moral and social norms of functioning, in other words, the person-level constructs I have been advocating as the locus at which mental disorders are and should be identified and classified. If we lose sight of these, and focus solely on differences at the neural level between people, we are left unable to say which differences *matter* for attributions of disorder. It is only by paying attention to what is going wrong at the person-level that it is possible to argue that an associated neural structure or function is indicative of pathology or problematic abnormality.

## Bringing psychiatry in from the cold

One of the major arguments in support of reconceptualizing mental disorders as brain disorders is the potential to make psychiatry seem more like other branches of medicine and reduce the stigma associated with having a ‘mental’ illness. Mental disorder does not necessarily mean ‘mental’, or in some sense ‘non-physical’, cause. Yet there remains a damaging hang up about having a mental disorder, which is perhaps a relic from the Cartesian distinction between mind and body. Patients with psychiatric diagnoses often feel poorly understood by clinicians, and fear that they are being judged as making up their symptoms, or that their pain, impairments and suffering are ‘all in the mind’. This phrase captures one of the key problems with the notion that there may be a psychological (rather than clear ‘physical’) basis for a person's symptoms: that because they are ‘mental’, they either do not really exist, or they should be able to be controlled or ameliorated by the person if they only tried hard enough. Symptoms can be perceived as moral failings and weakness of the will or of character, and also carry implications of malingering. The phrase ‘it's all in your mind’ continues to exert a strong moral judgement, despite the fact that we acknowledge the mind is indeed a very powerful thing. It is socially acceptable to cite stress as a cause of fatigue or hypertension, and psychological trauma is known to cause striking physiological responses. The placebo effect, a psychological phenomenon, can be extremely strong. The efficacy of cognitive behavioural therapy and other psychological interventions indicate the power of the mind to generate physical effects and alleviate distressing states ranging from anxiety, to the positive symptoms of schizophrenia. Yet the idea that one's symptoms may in some sense be psychological continues to generate stigma.

It is not clear, however, that classifying mental disorders as brain disorders, drawing psychiatry closer to other branches of medicine, and generating explanations of symptoms in terms of neuropathology or problems in brain functioning indicates the right direction of travel for classification, or indeed the means by which to reduce stigma. The further the scientific understanding of the brain advances, the more we are able to appreciate how complex the interrelations can be between genetics, development, brain structure and function, life events, interpersonal relationships and the social, cultural and economic environment. This suggests that the conceptual direction of travel for understanding mental disorders will need to be against the tide of conceptualization in biological, subperson-level terms. Rather than reducing mind to the functions and dysfunctions of the brain, it would be more astute to recognize the importance of person-level constructs in conceptualizing mental disorder, and use these as the anchor for psychiatric nosology, taking into account factors ranging from the broadly social to the narrowly genetic. As for stigma, perhaps increasing recognition that social and psychological factors are implicated in many physical health conditions may help mitigate against continued perceptions of a mind/body divide, and thus reduce the negative connotations of having a mental (as opposed to physical) disorder? It is beyond the scope of this paper to flesh out this consideration, but I raise it only to emphasize that reduction of mind to brain is not the only way to potentially positively affect social perceptions of mental disorder.

There are powerful and compelling sociological and institutional reasons to bring the fields of psychiatry and neurology closer together. After all, psychiatrists are first and foremost medically trained doctors, able to prescribe pharmaceuticals and investigate the pathology of the body. But this does not mean psychiatry, and the classification of mental disorders, ought to be collapsed into neurology and the study of brain diseases. If anything, the close relation between the mind and the body, the mental and the physical, and the psychiatrist's expertise in dealing with complex psychological and social issues, suggests neurology and other medical disciplines ought to draw closer to psychiatry.

As a multi-level science, psychiatry is actually ahead of the game in incorporating different levels of explanation, and different potential treatments targeting the brain, the person and their psychosocial environment. It has the scope to act as an intermediary between biomedicine, psychology and social work, and to remain agnostic about the relevant causal pathways that lead to disorder. Social determinants of health reveal these extrinsic factors are crucial to health although disciplinary specialities artificially separate them: every condition is both a physical and a mental disorder.

Jaspers argued that in order to understand psychopathology, we must not lose sight of attending to the patient's experience, not only for the purposes of treatment but also for keeping track of precisely what the phenomena are that we are interested in. A century later, amidst a flurry of exciting and revelatory discoveries about the workings of the brain, Jaspers’ tenet is a timely reminder. Conceptualizing mental disorders as brain disorders, even while giving due to their biopsychosocial context, would mistake what picks out a condition as a mental illness in the first place, that is, that something is going wrong at the person-level. The field of psychiatry may well be in the best position to accommodate the notion of multiple, multi-level causal pathways for disorders, and translate this understanding into good care and treatment for patients, if it does not make the conceptual error of assuming its subject matter necessarily needs to be characterized as pathologies of the brain.

## Footnotes

1 Editor's note: for a discussion of these broader questions and their relevance to practice, see the Conference Report section of this issue, which provides an overview of the workshop at which this paper was initially presented.

2 The social model of disability (e.g. Oliver & Sapey 9) rejects this view, which nonetheless predominates in medicine.

## References

[1] White PD, Rickards H, Zeman AZJ (2012). Time to end the distinction between mental and neurological illnesses. British Medical Journal.

[2] Szasz T (1961). The Myth of Mental Illness: Foundations of A Theory of Personal Conduct.

[b3] Jaspers K (1997). Allgemeine Psychopathologie. Berlin: Springer, 1913. English Translation of the 7th Edition: General Psychopathology.

[4] Maj M (2013). ‘Mental disorders as “brain diseases” and Jaspers’ legacy. World Psychiatry.

[5] Griesinger W (1845). Die Pathologie Und Therapie Der Psychischen Krankheiten.

[6] Boorse C, Humber JM, Almeder RF (1997). A rebuttal on health. What Is Disease?.

[7] Wakefield JC (1992). Disorder as harmful dysfunction: a conceptual critique of DSM-III-R's definition of mental disorder. Psychological Review.

[8] Greene J (2007). Prescribing by Numbers.

[9] Oliver M, Sapey R (2006). Social Work with Disabled People.

[10] Compton WM, Guze SB (1995). The neo-Kraeplinian revolution in psychiatric diagnosis. European Archives of Clinical Neuroscience.

[11] Stein DJ, Phillips KA, Bolton D, Fulford KWM, Sadler JZ, Kendler KS (2010). What is a mental/psychiatric disorder? From DSM-IV to DSM-V. Psychological Medicine.

[12] Insel TR, Wang PS (2010). Rethinking mental illness. JAMA: The Journal of the American Medical Association.

[13] A decade for psychiatric disorders (2010). Nature.

[14] Murphy D (2006). Psychiatry in the Scientific Image.

[15] Kim S, Thibodeau T, Jorgensen RS (2011). Shame, guilt and depression: a meta-analytic review. Psychological Bulletin.

[16] Murphy D, Zalta EN (2010). Philosophy of psychiatry. The Stanford Encyclopedia of Philosophy (Fall 2010 Edition).

[17] Bortolotti L, Broome MR (2009). Mental illness as mental: in defence of psychological realism. Humana Mente.

[18] Schwarz A (2012). http://www.nytimes.com/2012/10/09/health/attention-disorder-or-not-children-prescribed-pills-to-help-in-school.html.

